# Tympanal bone fracture in forensic practice

**DOI:** 10.1007/s00414-021-02682-0

**Published:** 2021-10-02

**Authors:** France Evain, Karl-Olof Lovblad, Tony Fracasso

**Affiliations:** 1 Forensic Pathology, University Center of Legal Medicine, Geneva University Hospitals and University of Geneva, rue Michel-Servet 1, CH-1211 Geneva 4, Switzerland; 2 Division of Neuroradiology, Diagnostic Department, Geneva University Hospitals, rue Gabrielle-Perret-Gentil 4, 1205 Geneva, Switzerland

**Keywords:** Tympanal, Fracture, Blunt, Head, Trauma

## Abstract

A tympanal bone fracture is an uncommon complication of head trauma and is mostly associated with a mandibular or petrous bone fracture. Upon reviewing the medicolegal literature, we could not find any publications on this topic. Tympanal bone fracture may lead to chronic complications (including external auditory canal stenosis and conductive hearing loss), with an important impact in both the medical and judiciary fields (e.g., chronic disabilities with loss of income). We decided to investigate the prevalence and mechanisms of tympanal bone fractures by means of a retrospective observational study on living victims who underwent head computed tomography after blunt head trauma and clinical forensic investigation at our center. We selected 159 cases of living victims with blunt head trauma (following an assault, traffic accident, or work accident) between January 2016 and December 2020. Re-examination of head imaging revealed 12 cases of tympanal bone fracture. Seven individuals showed cranial fractures involving the petrous bone (on the same side as the tympanal bone fracture). Three individuals had a temporomandibular fracture after a fall with chin impact. Only two victims exhibited an isolated tympanal bone fracture.

## Introduction

Tympanal bone fracture (TBF) is an uncommon complication of blunt head trauma and is mostly associated with a mandibular or petrous bone fracture [1–10]. It can lead to several complications, including external auditory canal (EAC) stenosis [2, 5, 11, 12] with conductive hearing loss [8, 11]. For this reason, it deserves proper medical follow-up and the attention of the forensic pathologist due to the risk of long-term disability and loss of income.

Intrigued by the observation of an isolated TBF after high-energy blunt chin impact in a victim who underwent clinical forensic investigation, we performed a review of the literature and found that this type of fracture is quite uncommon. Furthermore, we could not find any publication on this topic in the medico-legal literature. This is why we decided to investigate the prevalence and mechanisms of TBF by means of a retrospective observational study on living victims who underwent clinical forensic investigation at our center and head computed tomography (CT) after trauma during medical care at the hospital.

## Case presentation

A 20-year-old man was the victim of an assault on the street in Geneva (Switzerland) at night in July 2016. The victim had no memory of the assault (circumstantial amnesia). According to witnesses, three men tried to rob him, kicked him in the head, and escaped. Upon the arrival of paramedics, the young man was unconscious (Glasgow Coma Scale 8/15). He was intubated and transported to the Geneva University Hospitals for medical care. Shortly after, the public prosecutor ordered a clinical forensic investigation of the victim. The medicolegal investigation revealed two blunt wounds to the chin, multiple bruises on the left side of the face, and left otorrhagia. Head CT performed at the hospital for clinical purposes showed an isolated fracture of the left tympanal bone, interpreted as the consequence of blunt trauma with high-energy impact to the chin. The man was discharged from the hospital a few days later, without any known complications.

## Material and method

We performed an observational retrospective study. Firstly, we selected living victims who underwent radiological investigation and whose images were reviewed by the forensic radiological team of our center between 1 January 2016 and 31 December 2020 (*n* = 496). This imaging was performed during medical care, for clinical purposes. We excluded all the cases in which a head CT scan was not performed. Among the remaining cases, we excluded cases without a history of blunt head trauma. After this selection, 159 cases met both criteria of blunt head trauma and head CT scan imaging (regardless of the injection of a contrast agent). We re-examined all 159 head CT scans together with an experienced neuroradiologist in order to specifically search for TBF. We did not take into account the radiological reports (both clinical and forensic) available for the forensic investigation during the re-evaluation of the images performed by the forensic neuroradiologist for the purposes of the present study. In each case in which PBF was identified by the neuroradiologist, we collected information on the characteristics of the victim (gender and age), associated traumatic lesions, clinical features, and alleged traumatic scenario/mechanism from the medicolegal report.

## Results

Complete results are presented in Table 1.

**Table 1:** Characteristic of cases with tympanal bone fracture. *GCS*, Glasgow Coma Scale; *SAH*, subarachnoid hemorrhage; *SDH*, subdural hematoma; *CSF*, cerebrospinal fluid; *DAI*, diffuse axonal injury

Sex	Age	Side of TB fracture	Context of trauma	Clinical observations	Skin lesions	Associated craniofacial fractures	Proposed mechanism of TB fracture
M	20	Left	Assault: multiple kicks in the head	GCS 8/15, circumstantial amnesia, left otorrhagia, tympanal erythema, minimal left parietal SAH	Chin blunt wounds, multiple bruises of the left side of the face, external auditory canal disruption	_	Isolated, high-energy chin impact with mandibular kickback
M	38	Left	Assault: multiple kicks in the head	GCS 8–9/15, left otorrhagia, minimal right frontal SDH, right fronto-parietal SAH, fronto-polar intracerebral hemorrhages	Multiple bruises, blunt wounds, and abrasions of the face with sole shaped bruises of the forehead	Left fronto-temporal bone involving petrous bone, left ossicular disruption, left orbital floor	Petrous bone fracture extension
F	46	Bilateral	Accident and assault: fall with direct chin impact followed by kicks in the right side of the head	GCS 15/15, circumstantial amnesia, trismus	Chin blunt wound	Mandibular (symphysis and both condyles with anterior luxation), dental	High-energy chin impact with mandibular kickback and mandibular fractures
M	47	Right	Accident: fall from a height (4 m) with impact on the right side of the head	GCS 10/15, right otorrhagia, diffuse bilateral SDH and SAH	_	Multiple occipital and parietotemporal bone involving both petrous bones	Petrous bone fracture extension
M	21	Left	Assault: multiple kicks in the head	GCS 3/15, circumstantial amnesia, left otorrhagia, minimal right temporal SAH, occipital intracerebral hematoma, frontal and temporal intracerebral hemorrhages	Bruises and abrasion of the face	Left temporomandibular articulation	High-energy chin impact with mandibular kickback and mandibular fractures
M	51	Left	Accident: cyclist hit by a car (no helmet)	GCS 11/15, circumstantial amnesia, right SDH, bilateral SAH	Parietal posterior scalp blunt wound	Left fronto-temporo-parietal bones involving petrous bone	Petrous bone fracture extension
M	85	Right	Accident: fall after cardiac arrest (posterior impact)	GCS 3/15, right otorrhagia, hemotympanum, right SDH, fronto-temporal SAH, right temporal intracerebral contusion	Occipital abrasions and blunt wounds	Left petrous extending to right parietal bone, right carotid canal	Petrous bone fracture extension
M	85	Bilateral	Accident: fall from a height (7 m) with ventral impact	GCS 14/15, circumstantial amnesia, minimal factorial SDH	Right palpebral bruises, nasal bruise and abrasion, chin blunt wound	Mandibular (both subcondyles, right angle), bilateral temporomandibular articulation, maxilla, dental, nasal bones, left zygomatic arch	High-energy chin impact with mandibular kickback and mandibular fractures
M	40	Left	Accident: fall of a heavy object with impact on the left side of the head	GCS 11/15, circumstantial amnesia, left ear CSF discharge, left temporo-polar epidural hematoma, left temporo-polar intracerebral contusion	Bilateral orbital hematoma, hemorrhage of the left eyeball (hyposphagma)	Cranio-facial disjunction (LeFORT III), left temporal bone involving petrous bone, bilateral sphenoidal bone, multiple mandibular, left ossicular disruption	Petrous bone fracture extension
M	17	Bilateral	Accident: fall from a moving tractor	GCS 3/15, left hemotympan, left frontotemporo-parietal SDH, right fronto-temporal SAH, left fronto-temporoparietal SAH, DAI	Bruises of the left ear	Left parieto-temporal bone involving petrous bone, sphenoidal bones	Petrous bone fracture extension
M	32	Right	Assault: multiple kicks in the head	GCS 15/15, left otorrhagia, left parieto-temporooccipital epidural hematoma, bilateral cerebral contusions	Left temporo-parietooccipital hematoma, bruises of the left ear and periauricular region, abrasions of the left peri-auricular region and left cheek	Petrous bone (comminutive)	Petrous bone fracture extension
M	35	Right	Accident: fall from her height	GCS 15/15, left inferior limb fractures	Bruises and abrasions of the face, blunt wound of the chin	_	High-energy chin impact with mandibular kickback

Re-examination of the head CT scans of the 159 selected cases revealed 12 cases of TBF (7.55%), with six males and two females. The median age was 43 years (range 17–85 years). Nine unilateral fractures (six on the left side and three on the right side) and three bilateral fractures were observed. All the included cases were victims of high-energy blunt head trauma: four were victims of assault, with all of them characterized by kicks to the head; two were involved in a traffic accident, as a non-helmeted cyclist hit by a car and a fall from a moving tractor, respectively; two were victims of a work accident (fall from a 4-m height with impact on the right side of the head and the fall of a heavy object with impact on the left side of the head, respectively). Four cases accidentally fell to the ground: three with ventral impact, including one fall from a 7-m height, and one with posterior impact. It may be useful to underline here that in 10 out of 12 cases, both medical and forensic radiologists had detected TBF when present, as this was confirmed during the re-evaluation performed for the purpose of the present study (this last evaluation was done without knowledge of the previous radiological reports). However, in three cases, we discovered TBF that was described neither by the medical nor the forensic radiologist, even if their detection was not particularly challenging. This highlights the unfortunate lack of attention for such a fracture in clinical and forensic practice.

Forensic clinical examinations revealed otorrhagia on the side of the fracture in six victims, as well as blunt wounds, bruises, and abrasions of the scalp and/or the face in 11 victims. Specifically, chin blunt wounds were reported in four victims after either an assault or accidental fall with ventral impact. In two victims, hemotympanum on the same side as the TBF was present. Seven victims showed cranial fractures involving the petrous part of the temporal bone. Four victims presented a mandibular fracture, with one of them in association with multiple facial and cranial fractures. Ossicular disruption was found in two cases and was not associated with reported otorrhagia. Interestingly, only two victims exhibited an isolated TBF. No EAC stenosis was reported during the clinical observation.

## Discussion

The temporal bone is a component of the cranial vault and one of the thickest bones of the skull base. It consists of squamous, mastoid, petrous, and tympanal parts. The latter is a thin bone forming the floor and the anterior and posterior walls of the EAC [8, 13]. The tympanal bone is anatomically located right behind the temporomandibular joint (TMJ), where the mandibular condyles articulate with the temporal bone. In other words, the TMJ is anatomically separated from the EAC by the tympanal bone. Radiological assessment of the tympanal bone is not particularly challenging for the experienced radiologist, since it is easily recognizable. However, the evaluation of this tiny bone requires special attention from the clinical radiologist, as well as the forensic practitioner, and its visualization should not be forgotten. As a matter of fact, TBF can lead to chronic complications of importance in both the clinical and judiciary fields, notably EAC stenosis [1, 2, 5, 11, 12] with conductive hearing loss [8, 11] and subsequent loss of income for the victim. Furthermore, TBF rarely causes cholesteatoma formation [11, 14], which may require surgical treatment.

**Fig. 1:**
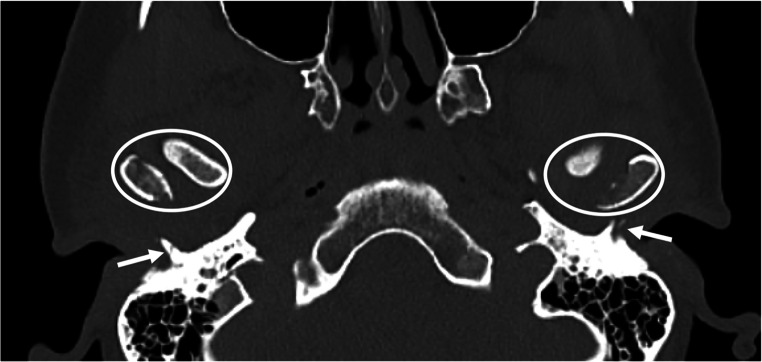
Computed tomography showing bilateral tympanal bone fracture (arrows) associated with bilateral fracture of mandibular condyle with internal luxation (circles). The victims fell on the ground with an impact on the chin

**Fig. 2:**
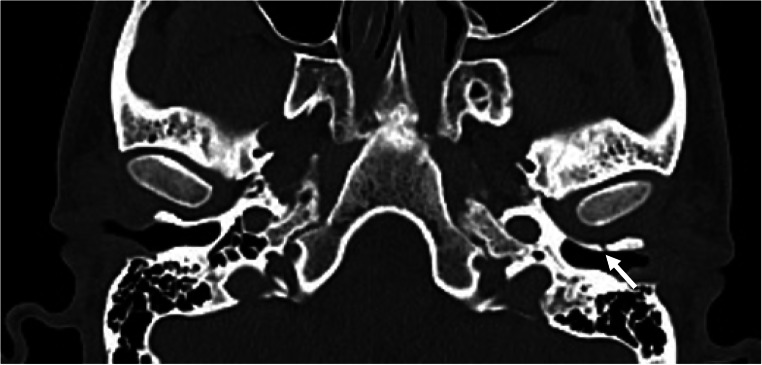
Computed tomography showing isolated left tympanal bone fracture (arrow). The victim received multiple kicks in the head during an assault

TBFs are uncommon complications of head trauma [1–3]. In the rather scarce scientific literature on this topic, the most reported mechanism is high-energy mandibular trauma (with impact to the chin), during which the mandibular head is retropulsed into the glenoid fossa of the TMJ strongly enough to overpass the resistance of tendons, ligaments, and cartilage, resulting in impaction and fracture of the tympanal bone [2, 5, 8, 10–12, 15]. TBFs are usually associated with mandibular fractures (Fig. 1). However, it can rarely be isolated (Fig. 2) [3, 10].

Victims of blunt head trauma often undergo radiological examination during medical care at the hospital in order to explore cranial and intracranial traumatic injuries. In the forensic centers of the French-speaking part of Switzerland, we systematically perform a re-examination of the clinical imaging when the prosecutor requires a forensic examination of these victims. This re-examination, which is complementary to the forensic clinical investigation, is performed by our forensic radiological team. It offers an assessment of radiologically visible traumatic lesions from a forensic standpoint (e.g., subcutaneous hemorrhage at the site of impact) in order to better understand the trauma mechanism and/or its consequences. It also provides a useful iconography of the lesions to be presented to the public prosecutor.

Typical clinical manifestations of acute TBF include otorrhagia [2, 8, 10, 11, 15] secondary to the skin lesion of the affected EAC and ossicular disruption (given the close anatomy of both structures) with hypoacusis [2, 8, 16] and limitation of mouth opening [2, 8, 11, 16]. However, as we can see in the present study, such symptoms are not always present or clinically detected and TBF should be searched for even without symptoms.

Long-term complications of TBF include formation of scar tissue leading to EAC stenosis [2, 11, 12], which may eventually lead to conductive hearing loss requiring surgical management in order to open the EAC. Moreover, such stenosis may impede the outward migration of stratified epithelium of the middle ear. After years, the trapped epithelium can eventually result in a post-traumatic cholesteatoma [12, 14]. Other proposed mechanisms for the development of post-traumatic cholesteatomas include the entrapment of the stratified epithelium in the fracture line and the traumatic implantation of tympanic membrane skin into the middle ear [14]. Post-traumatic cholesteatomas can remain in the EAC, invade the tympanic membrane and middle ear, invade the petromastoid cells, or extend to other parts of the temporal bone [14]. In severe cases, surgical resection is mandatory. Furthermore, if left untreated, EAC stenosis can induce conductive hearing loss [11]. Therefore, TBF requires a proper diagnosis, medical survey, and occasionally an otologic treatment (packing and/or stent of the EAC) in order to prevent complications [11, 12, 14].

Given the rare but real risk of long-term and preventable complications (hearing loss and cholesteatoma), which is potentially debilitating for the victim and demands further medical care, forensic practitioners must be alert and should proactively look for TBF in cases of craniofacial trauma.

Our observations based on 159 victims of high-energy blunt head trauma confirm that PBFs are uncommon complications of craniofacial high-energy blunt trauma. Isolated PBFs are indeed rare but still can occur after a high-energy impact to the chin. Forensic practitioners should be attentive and look for TBFs on head CT scans, especially (but not exclusively) in cases of mandibular and/or cranial trauma, even without classical symptoms (such as otorrhagia). Indeed, victims can undergo potentially severe long-term complications, such as chronic EAC stenosis (with conductive hearing loss) and cholesteatoma.

## Conclusion

TBF is uncommon after high-energy blunt head trauma, and its assessment requires special attention. The mechanisms involved are mandibular head kickback into the articular fossa following high-energy impact on the chin (usually associated with temporomandibular fracture) and extension of a petrous bone fracture to the tympanal bone. Detection of TBF on head CT is not particularly challenging for a radiologist, but it requires special attention and should not be forgotten. Indeed, although rare, TBF may lead to chronic preventable complications, such as EAC stenosis with conductive hearing loss and/or cholesteatoma, which may require surgical management. These complications can be important, notably in the judiciary field due to the risk of long-term disability and potential loss of income. Unfortunately, we have no information about the follow-up of the victims affected by TBFs in our study. The tracking of auditory complications of these fractures could be subject to further study.
